# The inhibition of 45A ncRNA expression reduces tumor formation, affecting tumor nodules compactness and metastatic potential in neuroblastoma cells

**DOI:** 10.18632/oncotarget.14138

**Published:** 2016-12-24

**Authors:** Ilaria Penna, Arianna Gigoni, Delfina Costa, Serena Vella, Debora Russo, Alessandro Poggi, Federico Villa, Antonella Brizzolara, Claudio Canale, Andrea Mescola, Antonio Daga, Claudio Russo, Mario Nizzari, Tullio Florio, Paola Menichini, Aldo Pagano

**Affiliations:** ^1^ Department of Experimental Medicine (DIMES), University of Genova, Genova, Italy; ^2^ Department of Internal Medicine (DIMI), University of Genova, Genova, Italy; ^3^ IRCCS-AOU San Martino-IST, Genova, Italy; ^4^ Department of Laboratory Medicine and Advanced Biotechnologies, IRCCS-ISMETT (Istituto Mediterraneo per i Trapianti e Terapie ad Alta Specializzazione), Palermo, Italy; ^5^ Nanophysics Unit, Italian Institute of Technology, Morego, Genova, Italy; ^6^ Department of Health Sciences, University of Molise, Campobasso, Italy; ^7^ Center of Excellence for Biomedical Research (CEBR), University of Genova, Genova, Italy

**Keywords:** neuroblastoma, non-coding RNA, GTSE1, tumorigenesis, metastasis

## Abstract

We recently reported the *in vitro* over-expression of 45A, a RNA polymerase III-transcribed non-coding (nc)RNA, that perturbs the intracellular content of FE65L1 affecting cell proliferation rate, short-term response to genotoxic stress, substrate adhesion capacity and, ultimately, increasing the tumorigenic potential of human neuroblastoma cells. In this work, to deeply explore the mechanism by which 45A ncRNA contributes to cancer development, we targeted *in vitro* and *in vivo* 45A levels by the stable overexpression of antisense 45A RNA.

45A downregulation leads to deep modifications of cytoskeleton organization, adhesion and migration of neuroblastoma cells. These effects are correlated with alterations in the expression of several genes including GTSE1 (G2 and S phase-expressed-1), a crucial regulator of tumor cell migration and metastatic potential. Interestingly, the downregulation of 45A ncRNA strongly affects the *in vivo* tumorigenic potential of SKNBE2 neuroblastoma cells, increasing tumor nodule compactness and reducing GTSE1 protein expression in a subcutaneous neuroblastoma mouse model. Moreover, intracardiac injection of neuroblastoma cells showed that downregulation of 45A ncRNA also influences tumor metastatic ability. In conclusion, our data highlight a key role of 45A ncRNA in cancer development and suggest that its modulation might represent a possible novel anticancer therapeutic approach.

## INTRODUCTION

Non-coding (nc)RNA are widely recognized as crucial regulators of several physiological and pathological processes [1].

We recently reported that the expression of 45A [2], a pol III-transcribed ncRNA, perturbs the intracellular content of FE65L1 (APBB2, amyloid beta (Aβ) precursor protein-binding, family B, member 2, NP_004298.1), a protein potentially involved in the pathogenesis of Alzheimer disease (AD) due to its binding to amyloid precursor protein (APP, P05067) [3], [4]. Generation of the Aβ peptide and APP C-terminal fragment gamma are potentiated by the overexpression of FE65L1 and decreased if an alternatively spliced, different form of the protein is synthesized [2]. Interestingly, in our studies we demonstrated that in neuroblastoma cells FE65L1 exerts a primary role in cell cycle regulation and genomic surveillances. Indeed, studying the effects of the 45A ncRNA overexpression on FE65L1 splicing and the consequent Aβ processing, we found, besides an altered amyloid release, an enhanced cell proliferation and, ultimately, an increase of the tumorigenic potential of tumor-derived cells. At the molecular level, this finding was supported by multiple evidences including the restriction of G2 and G2/M transition induced in 45A-overexpressing cells, the upregulation of different genes that promote cell proliferation (and the downregulation of those involved in differentiation and cell cycle arrest after DNA damage), and a short-term greater sensitivity to different genotoxic agents. Besides the increased tumorigenic potential *in vitro*, we also showed that 45A-overexpressing cells exhibit an enhanced potential to develop tumor *in vivo*, confirming the active role of 45A ncRNA in the determination of the malignant phenotype of NB cells [2].

Although in our previous work we showed that 45A ncRNA overexpression affects DNA damage response, cell proliferation and tumorigenesis [2], the mechanism by which this ncRNA contributes to tumor development still needs to be explored as 45A ncRNA targeting might constitute a novel approach to hinder tumorigenesis. In detail, our results were obtained using a highly malignant NB cell line and this might represent a potentially relevant model for the therapy of high risk NB.

NB is a pediatric tumor characterized by remarkable cellular heterogeneity. Among different NB stages, high risk NBs are characterized by poor prognosis and this cancer still accounts for about 15% of all pediatric cancer deaths [5].

In this work we explore the possibility to inhibit NB cell tumorigenic potential reducing the activity of 45A ncRNA *in vitro* and *in vivo*, through the stable overexpression of an anti-45A RNA. We show that deep modifications of cytoskeleton, cell adhesion and migration are induced after 45A downregulation. We demonstrate that this phenotype is consequent of altered expression of specific genes among which the downregulation of GTSE1 (G2 and S phase-expressed-1, NM_016426) plays a crucial role in the alteration of cell migration and, possibly, metastatic potential. Notably, we demonstrated that 45A ncRNA downregulation in malignant NB cells strongly affects tumor progression, increasing tumor nodule compactness and reducing GTSE1 protein expression (Q9NYZ3) in a mouse subcutaneous NB model. Interestingly, intracardiac injection of NB cells showed a peculiar tumor spreading indicating a key role for 45A ncRNA in the formation of metastasis. Therefore, our data suggest the possible control of 45A ncRNA expression as novel anticancer therapeutic approach.

## RESULTS

### The downregulation of 45A ncRNA synthesis impairs cell proliferation

**Figure 1:**
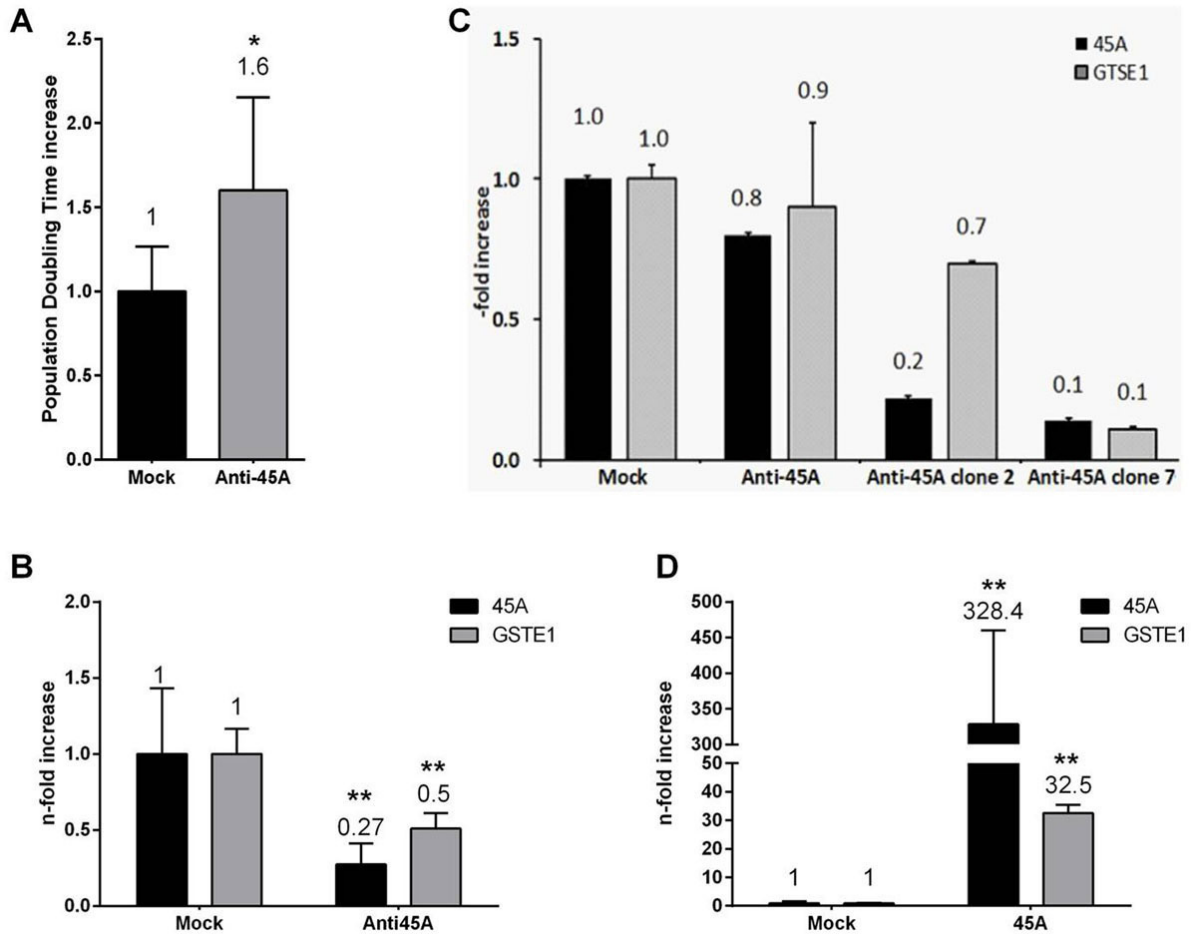
The downregulation of 45A ncRNA delays cell proliferation and induces cytogenetic features changes **A**. Population doubling time increased of SKNBE2 Mock and Anti45A permanently transfected cell lines. Results are normalized to Mock control. Data represent mean ± SD of 7 replicates (*p < 0.05). **B**. 45A (black bars) and GTSE1 (grey bars) expression level analyzed by Real-Time RT-PCR in SKNBE2 cell lines. Data represent mean ± SD (**p < 0.01). **C**. 45A and GTSE1 expression level in different SKNBE2 cell clones permanently transfected with Anti-45A plasmid construct. **D**. 45A and GTSE1 expression level in Mock and 45A-overexpressing SKNBE2 cells.

In order to investigate the effects of 45A ncRNA downregulation on the NB tumorigenic potential, we first generated several clones of SKNBE2 cells permanently transfected with a plasmid harboring 45A RNA transcribed portion in antisense (AS) configuration, whose transcription is driven by its natural pol III type 3 promoter. Among them, for the next experiment, we chose a SKNBE2 clone in which 45A ncRNA level was decreased by 73% (p<0.0001, Figure 1B). In particular, we compared the proliferation rate of this SKNBE2 Anti-45A clone, with Mock-transfected cells, by direct determination of population doubling time. We found that anti-45A RNA synthesis, and the consequent downregulation of 45A ncRNA, causes a significant increase of the population doubling time (p= 0.0266, Figure 1A).

In addition, we have detected an Anti45A-dependent decrease of cell proliferation rate in a non-Myc-N amplified neuroblastoma cell line, SH-SY5Y, evidencing that the results reported in this work can be obtained in different neuroblastoma cells (data not shown).

Thus, in agreement with the observation that the increased expression of 45A ncRNA promotes cell proliferation and increases tumorigenic potential, we show that the inhibition of its expression significantly limits the proliferation rate.

### The silencing of 45A impacts on DNA damage response

We previously showed that the 45A expression significantly affects cell cycle progression after the exposure to different genotoxic agents, and confers a short-term sensitization toward doxorubicin and methylmethanesulfonate (MMS). However, compared to Mock cells, 45A-overexpressing cells did not accumulate a significant level of DNA damage, as determined by micronuclei and multinucleated cells induction [2].

**Figure 2:**
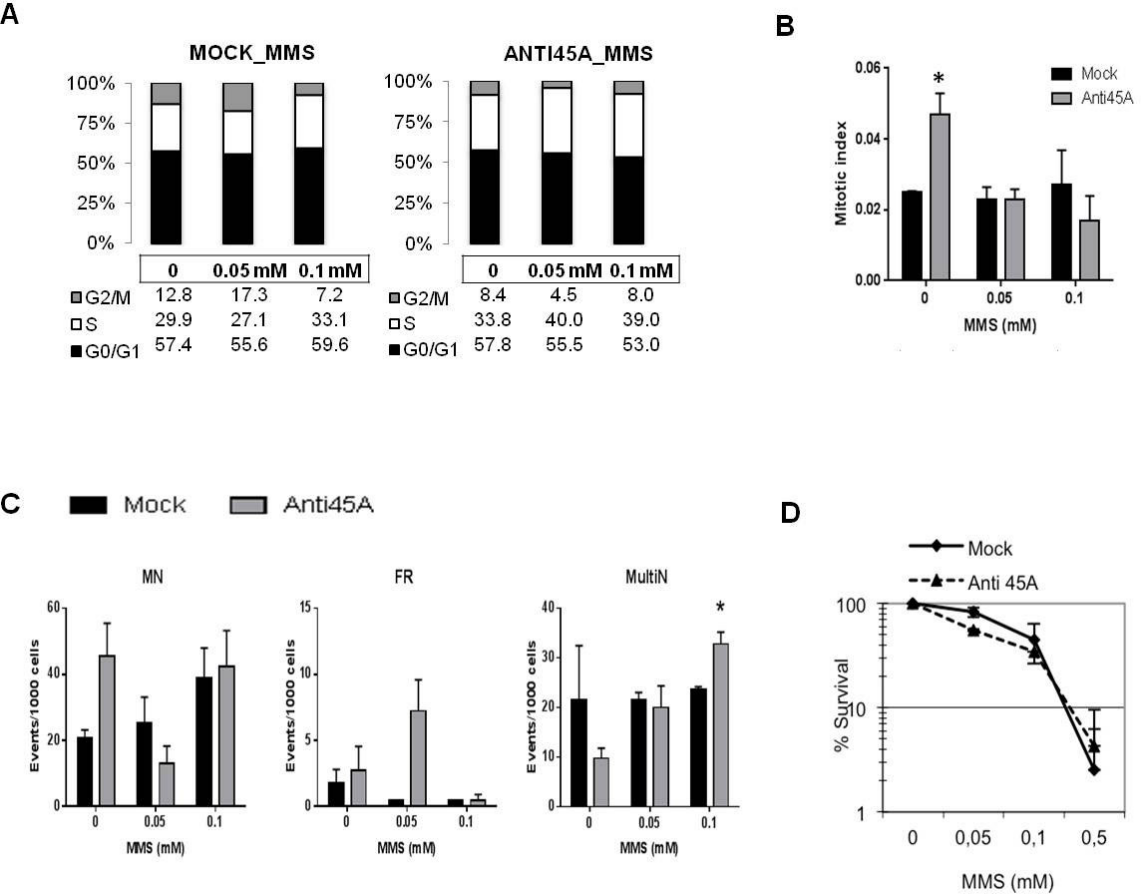
The downregulation of 45A ncRNA impacts on DNA damage response **A**. Distribution of HEK-293 Mock and Anti45A cell populations in cell cycle phases after MMS treatments and **B**. Mitotic Index representation. **C**. Evaluation of Micronuclei (MN), Fragmented cells (FR), Multinucleated cells (MultiN) and **D**. survival rate in HEK-293-Mock and HEK-293-Anti45A before and after MMS treatment. Data represent mean ± SD. One star (*) indicates p < 0.05, two-tailed Student's test.

In the light of these results, we investigated whether the delayed cell proliferation rate observed in Anti-45A expressing cells might affect the distribution among cell cycle phases, the accumulation of DNA damage, and cell survival following MMS treatment (Figure 2); to this aim, we used HEK-293-Anti45A and HEK-293-Mock cells as experimental model. Treatment with MMS affected the cell cycle progression in a dose-dependent manner in all the cells, although this effect was rather modest. However, comparing MMS effect in Anti-45A expressing and Mock cells, we observed that 0.05 mM MMS caused an increase in the percentage of Anti-45A cells entering in the S-phase, concomitantly with the decrease of the percentage of cells in G2/M. Conversely, the percentage of G2/M cells slightly increased in Mock cells (Figure 2A). At 0.1 mM MMS, similar cell cycle profiles were found in both cell lines, suggesting that a higher level of MMS-induced damage may trigger a different response, likely due to the activation of different DNA repair pathways. Nevertheless, the mitotic index was reduced following MMS treatment of Anti-45A cells, in agreement with a decreased G2/M fraction (Figure 2B).

The induction of DNA damage, detected as micronuclei (MN), fragmented nuclei (FR), and multinucleated cells (MultiN), was evaluated. While MN induction did not differ significantly between Mock and Anti45A expressing cells (Figure 2C), more FR nuclei at the lower 0.05 mM MMS concentration and a higher number of MultiN cells at 0.1 mM MMS were found in 45A-silenced compare to Mock cells (Figure 2C). Accordingly, the survival curve calculated following MMS exposure revealed a greater sensitivity of Anti45A cells than Mock at the 0.05 mM MMS (Figure 2D). A higher amount of MN, FR and MultiN cells in Anti-45A compared to Mock cells were also found after UV exposure (Supplementary Data 1A); such an impaired processing of UV-induced DNA damage was reflected in a greater sensitivity of Anti-45A than Mock cells toward UV light (Supplementary Data 1B).

More FR nuclei at the lower 0.05 mM MMS concentration and a higher number of MultiN cells at 0.1 mM MMS were found in 45A-silenced cells compared to Mock cells (Figure 2C). Accordingly, the survival curve calculated following MMS exposure revealed a greater sensitivity of Anti45A than Mock cells after treatment with 0.05 mM MMS (Figure 2D).

Altogether these results indicate that the silencing of 45A ncRNA perturbs the progression through the cell cycle in the presence of DNA damage caused by MMS, a genotoxic agent able to trigger different DNA repair pathways such as the base excision repair (BER). Such a perturbation correlates with a higher burden of genomic damage.

### The 45A ncRNA downregulation significantly affects the expression of cell division- and cell morphology-related genes

**Figure 3:**
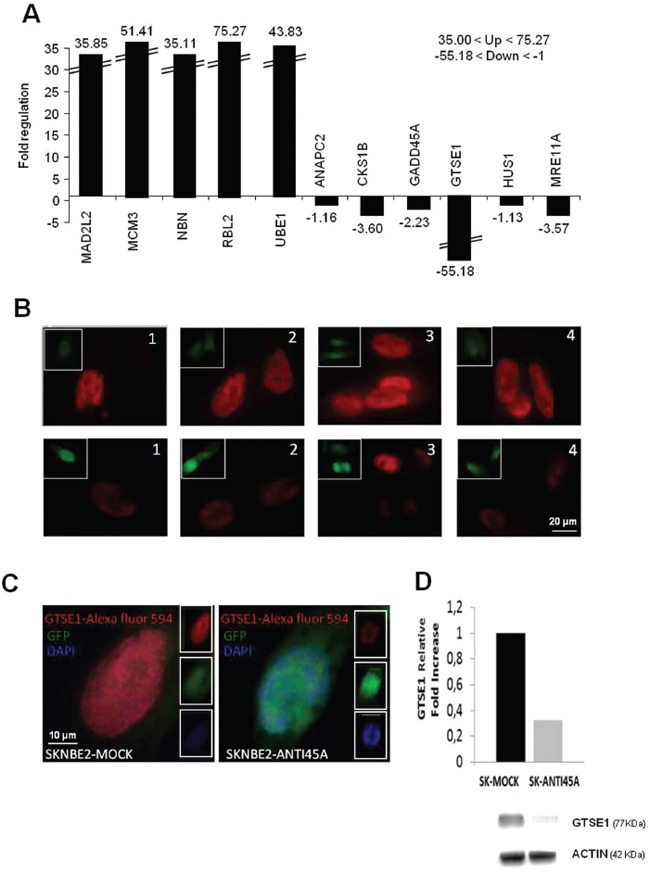
The downregulation of 45A ncRNA modulates the expression of specific cell cycle related genes **A**. Expression profile of genes involved in cell cycle progression and in cell cycle control in HEK-293-Anti45A/HEK-293-Mock cells; these genes have been considered arbitrarily imposing a cut-off (+35<up-regulated genes<+75.27 and -55.18<down-regulated genes<-1). **B**. 1-4: Representative GTSE1 IF on SKNBE2-Mock (CTR) (upper panels) and SKNBE2-Anti45A (lower panels). **C**. High magnification of single cell stained with Red-anti-GTSE1, Blue-Dapi and Green-GFP. **D**. GTSE1 Western blotting analysis of SKNBE2-Mock and SKNBE2-Anti45A cells. Equal loading of proteins was ensured by β-actin expression. Cropped gel retains important bands of GSTE1 and β-actin.

Since the downregulation of 45A ncRNA affects cell proliferation, we hypothesized that this phenomenon might involve the modulation of the expression of genes involved in cell cycle control. To test this hypothesis, we measured the simultaneous expression of 84 genes involved in cell cycle control by cell cycle-specific Real-Time low density array (Human Cell Cycle RT^2^ *Profiler* ^TM^ PCR Array), using HEK-293-Anti45A and HEK-293-Mock cells. By arbitrarily imposing appropriate cut-off values (+35<y<+75.27 gene expression for the upregulated genes and -55.18<y<-1 gene expression for downregulated genes), we found 5 genes whose expression was significantly upregulated by 45A ncRNA downregulation and only 1 (GTSE1) strongly downregulated (Figure 3A). This result was confirmed by Real-Time RT-PCR in SKNBE2 cells (GTSE1 expression was decreased by 50%, p=0.0011, Figure 1B) that showed a direct correlation between the expression level of 45A and GTSE1 (Figures 1B and 1C). To further strengthen the correlation between 45A/GTSE1 expression and to unequivocally demonstrate that the effects here observed in 45A downregulated cells are not restricted to the SKNBE cell clone used, (and maybe due to a peculiar genomic integration site of the transgene that could lead to an idiosyncratic behaviour of this clone), we investigated the possible correlation of the expression level of 45A ncRNA and GTSE1 in 3 independent HEK-293 clones permanently transfected with Anti-45A ncRNA construct and expressing 45AncRNA at different levels. As reported in Figure 1C, we demonstrated the strong correlation (Pearson correlation index r = 0.84) between the downregulation of 45A ncRNA and the decrease of GTSE1 expression. In addition, to further strengthen this correlation, we measured by Real-Time RT-PCR 45A ncRNA and GTSE expression in Mock and 45A-overexpressing cells. The results showed that overexpression of this ncRNA leads to up-regulation of GTSE1 (Figure 1D).

Interestingly, we also observed in Anti-45a overexpressing cells the upregulation of a subset of genes involved in the maintenance of chromosome integrity and proper cell division in response to DNA damage (Supplementary Data 2). However, the remarkable downregulation of GTSE1 drew our attention as we found this gene significantly upregulated in 45A-overexpressing cells (data not shown). Interestingly, GTSE1 has been recently described as a microtubule protein involved in cell migration regulation and in metastasis [6], [7]. Thus, to corroborate Real-Time RT-PCR results, we performed immunofluorescence (IF) staining and Western blot analysis for GTSE1 in SKNBE2-Mock and SKNBE2 Anti-45A cells, detecting a significant decrease of the cytoplasmic fraction of GTSE1 protein in SKNBE2 Anti-45A compared to Mock cells (Figures 3B and 3D). Therefore, these results suggest a link between an impairment of cell cycle progression driven by 45A ncRNA downregulation and specific changes of the cytoskeleton associated to this condition.

### The inhibition of 45A affects cell morphology and migration potential

The induction of nuclear fragmentation and particularly of multinucleated cells drew our attention.

**Figure 4:**
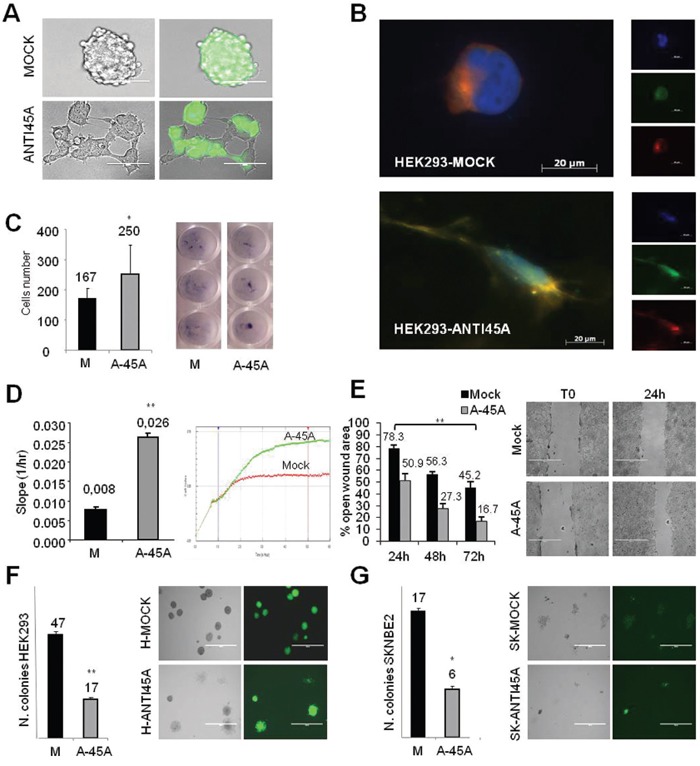
Cell morphology and migration potential after inhibition of 45A ncRNA **A**. Morphological analysis of colonies grown in adhesive condition of HEK-293-M and HEK-293-Anti45A cells. Right panels: GFP-positive colonies, as shown in the overlay picture. **B**. Morphological analysis of HEK-293-Mock and HEK-293-Anti45A permanently transfected cell lines, by ApoTome Microscope Technique from Carl Zeiss. Blue=DAPI, green=GFP, red=α-tubulin. **C**. Adhesion properties of HEK-293-Mock and HEK-293-Anti45A permanently transfected cell lines. Adherent cells were stained by methylene blue staining (right panel) and quantified by calculating the mean of cells counted in 3 independent wells (left panel). Data represent mean ± SD (*p < 0.05). **D**. Migration assay: using the xCELLigence RTCA DP Instrument (Roche), migratory capability of HEK-293-Mock and HEK-293-Anti45A cells has been quantified by the slope of the migration curve calculated by the RTCA 1.2 Software. Data represent mean ± SD (**p < 0.01). **E**. Wound healing assay: left panel shown quantitative analysis of open wound area percentage at different time point compared to T0 in HEK-293-Anti-45A and HEK-293-Mock cells; right panel shown representative image of open wound area (4x magnification). Results are representative for three independent experiments (mean ± SD, **p < 0.01). **F**. Capability of HEK-293 and G. SKNBE2 cells to form colonies in methylcellulose. Data represent mean ± SEM (*p < 0.05).

Multinucleation, characterized by the accumulation of nuclei within the cytoplasm of a single cell, may result from defects involving centrosomes, spindle checkpoints or cytokinesis and can be associated with delay in cell cycle progression [8]. Thus, it is likely that the formation of multinucleated cells is due to an impaired expression of genes coding for components of the cytoskeleton [9]. The observation that Anti45A cells exhibit a peculiar morphology, not detected in Mock- or 45A-overexpressing cells, is in agreement with this hypothesis. As shown in Figure 4A, colonies formed by HEK-293-Mock cells are rounded and poorly adherent whereas those originated by HEK-293-Anti-45A cells exhibit a spread, strongly adherent shape. As expected, once these cells were challenged by anti-tubulin IgGs, we found, at single cell level, remarkable differences of the cytoskeleton organization that confers the characteristic stretched form (Figure 4B). These observations suggest that Anti-45A cells might be particularly adherent due to specific changes at the level of cytoskeleton structure. To investigate cell adhesion properties, we seeded HEK-293-Anti-45A and HEK-293-Mock cells on uncoated dishes. After 3h we centrifuged the plates upside down and stained them with methylene blue. HEK-293-Anti-45A had a strongly adherent phenotype as their rate of detachment during centrifugation was significantly reduced as compared to the Mock cells (Figure 4C).

Next, we tested the influence of 45A downregulation on cell migration potential by two different approaches. By xCELLigence RTCA, a technology that allow the dynamic recording of the entire cell migration process in real time, we demonstrated that HEK-293-Anti-45A cellsshow a significantly increased migration than Mock cells (Figure 4D). This finding was further strengthening by a wound-healing assay, in which cells are allowed to fill the gap up to 24 h after seeding (Figure 4E). We observed an increased ability of HEK-293-Anti-45A to fill the wound, whereas HEK-293-Mock cells left a more open gap in the same time frame.

Considering that the overexpression of 45A ncRNA increases the tumorigenic potential of cancer cells and that its downregulation brings to an increased adhesiveness that, in principle, might be considered as decreased capability to growth in non-adherent conditions, we hypothesized that Anti-45A cells might exhibit a decreased tumorigenic potential. To verify our hypothesis, we tested the ability of the cells to form colonies in non-adhesive conditions (methylcellulose). We found that SKNBE2-Anti45A cells are characterized by a reduced capability to form colonies in non-adhesive conditions (Figures 4F and 4G).

In conclusion, these data show that the 45A ncRNA downregulation confers the cell a less rounded shape more prone to adhere to the substrate and migrate, but less suitable to form colonies in methylcellulose, and thus possibly less efficient in the colonization of tissues and in the formation of metastasis.

### The downregulation of 45A ncRNA expression affects cell stiffness

**Figure 5:**
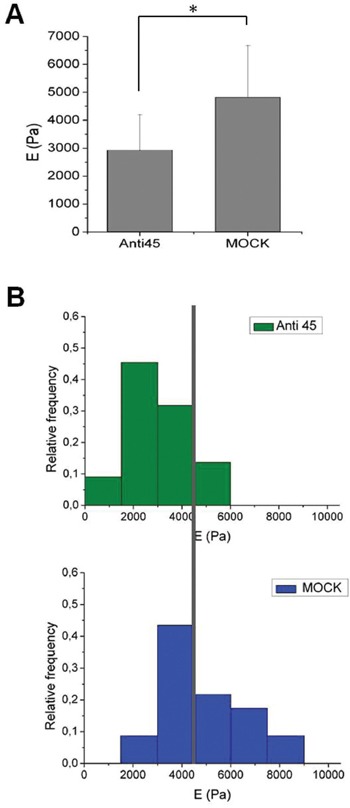
Quantitative analysis of cell stiffness **A**. Values of E calculated on Anti45A and Mock cells. Mock cells are significantly stiffer with respect to Anti 45 cells (*p<0.05). The error bars represent the standard deviation (SD) with respect to the mean value. **B**. Distributions of E calculated for Anti45 and Mock cells. Less than 15% of Anti45 cells have a Young's modulus higher than 4.5 kPa (the gray vertical line is positioned at this value), while almost 50% of Mock cells are exceeding this value of E.

The altered ability to adhere to the substrate of 45A-downregulated cells, their altered migration properties, the peculiar shape of their colonies, together with the fact that they exhibit a downregulation of a cytoskeleton-associated protein, may suggest that 45A ncRNA is involved in the determination or maintenance of cell stiffness. In order to address this issue, indentation experiments were performed by atomic force microscopy (AFM) as described (see Materials and Methods). The Young's modulus was calculated per each cell, mediating the values obtained by applying the Hertz's contact theory on all the FI curves acquired on the single cell (1024 FI curves per cell). Figure 5A shows the average values of E (mean ± SD) for the different cell types (Anti45 n=22; Mock n=23) whereas the distributions of the mean values of E are reported in Figure 5B. The results obtained for both cell types were significantly different (p<0.05), demonstrating that a reduced activity of 45A ncRNA leads to a decreased stiffness and an increased flexibility of the cells. These results suggest a different behaviour of 45A-downregulated cells in metastatic colonization and are in agreements with previous findings obtained on different kinds of cancer cells [10]–[15].

### Expression of Anti-45A slow down tumor growth *in vivo*

**Figure 6:**
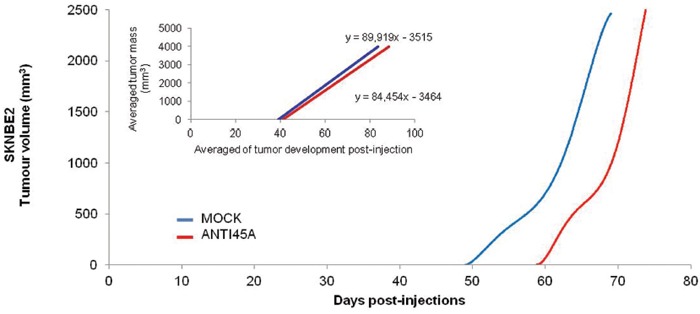
Anti45A ncRNA expression decreases cancer progression in vivo Tumor development function by the polynomial interpolation of SKNBE2 tumor nodules. The inset of the figure represents the linear regression of the averaged progression rate.

To assess whether 45A ncRNA downregulation affects tumor formation *in vivo*, we injected subcutaneously 3×10^6^ Anti45A-overexpressing SKNBE2 cells (and the correspondent Mock population) in a pool of 20 NOD-SCID mice, and evaluated possible differences of growth/tumor progression rate. As shown in Figure 6,40 days after the injection, 4/5 (80%) of the SKNBE2-Mock-injected mice developed a tumour nodule ≥5 mm whereas only 2/5 (40%) of the SKNBE2-Anti45A-injected mice developed nodules. In addition, we observed a slight reduction of the average growing rate of nodules in SKNBE2-Anti45A- compared to the SKNBE2-Mock- injected mice (84 454 and 89 919 mm^3^/day, respectively; inset in Figure 6).

Therefore, these results suggest that the 45A ncRNA downregulation does not affect tumour initiation but rather impacts on the tumour growth rate.

### The downregulation of 45A ncRNA leads to an increased compactness of tumor nodules and to reduced level of KI-67 and GTSE1 cancer markers

**Figure 7:**
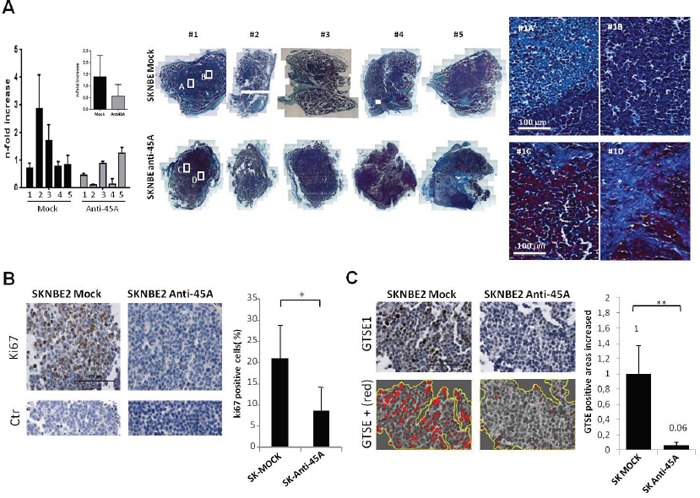
45A ncRNA down-regulation increased tumor nodule compactness and collagen fibers organization **A**. Representative light microscopy images of Mallory's Trichrome stained section (10x magnification reconstruction and 40x magnification particulars) and 45a expression level determined by Real-Time RT-PCR in SKNBE2 tumor nodules. Data represent mean ± SD. The averaged results for each group are also reported in the inset (p=0.26). **B**. Representative images at high magnification of KI-67 Immunohistochemical staining in SKNBE2-Anti45A and in SKNBE2-Mock tumour nodules sections. Lower panels are representative of negative control staining for KI-67 (CTR) (scale bar 100 μm). The quantification of KI-67 DAB positive cells in SKNBE2-Anti45A and SKNBE2-Mock tumour nodules sections is reported. Data represent mean ± SD (*p < 0.05). **C**. Representative images at high magnification of GTSE1 immunohistochemical staining in SKNBE2-Anti45A and in SKNBE2-Mock tumour nodules sections. Lower panels report the GTSE1 positive area selected from the above panel using ImageJ (scale bar 100 μm). The quantification of GTSE1 DAB positive cells in SKNBE2-Anti45A and SKNBE2-Mock tumour nodules sections is reported as average percentage from different mice (mean ± SD, **p < 0.01).

In the light of the significant variations of cell morphology, adhesion, migration and *in vivo* tumor growth ability, we postulated possible differences in the structural features of tumor nodules. In order to better identify the histological differences, we analyzed tumour nodules by Mallory's trichrome staining, which evidence blue stromal tissue and red cellular components. The comparison of SKNBE2 histological sections showed that 45A downregulated nodules exhibited more compact collagen fibers resulting in a more evident cellular component than in Mock nodules. Differently, Mock tumor nodules showed the cellular component more dispersed in connective fibrous stroma, with a loss of the fibrous organization in which the cell elements are spread (Figures 7A and 7B). In agreement with this observation, the analysis of 45A ncRNA expression in the nodules, by Real-time RT-PCR, revealed an inverse correlation between the 45A ncRNA expression level and tumor nodules compactness (Figure 7A). Altogether these results are compatible with a peculiar intercellular adhesion by activation of specific genetic programs for cell-cell contact in 45A downregulated cells. Thus, we speculate that the downregulation of 45A ncRNA would reduce SKNBE2 ability to escape from the primary tumor, leading to an altered potential to generate metastasis.

Next we performed immunohistochemical analysis of KI-67 protein (P46013), a marker associated to cell proliferation. We found lower levels of KI-67 expression in tumor nodules obtained from mice injected with Anti-45A cells (Figure 7B) (see also Supplementary Data 3). Notably, the amount of KI-67 positive cells in different mice correlated to the expression level of 45A ncRNA in the same tumour nodule (see Figure 7A). These results are in keeping with a reduced proliferation rate of cells from Anti45A tumor masses driven by a low expression of the ncRNA.

In the light of the increased compactness of Anti-45A tumor nodules, we hypothesized a correlation between the level of GTSE1 protein and the invasiveness/migration capability dependent on microtubule organization. To verify this hypothesis, we analyzed GTSE1 protein level in tumor nodules from Mock and Anti-45A mice in immunohistochemistry experiments. We found that in Anti-45A tumour nodules GTSE1 expression is significantly reduced with respect to Mock tumor nodules (Figure 7C) (see also Supplementary Data 4, 5 and 6).

Since GTSE1 is an important player in cell migration and its dysregulation was associated with increased invasive potential in breast cancer [6], our results suggest a possible reduced aggressiveness or metastatic potential of 45A-downregulated cells pointing toward a putative anticancer activity of this ncRNA.

### 45A ncRNA plays a key role in the formation of metastasis

**Figure 8:**
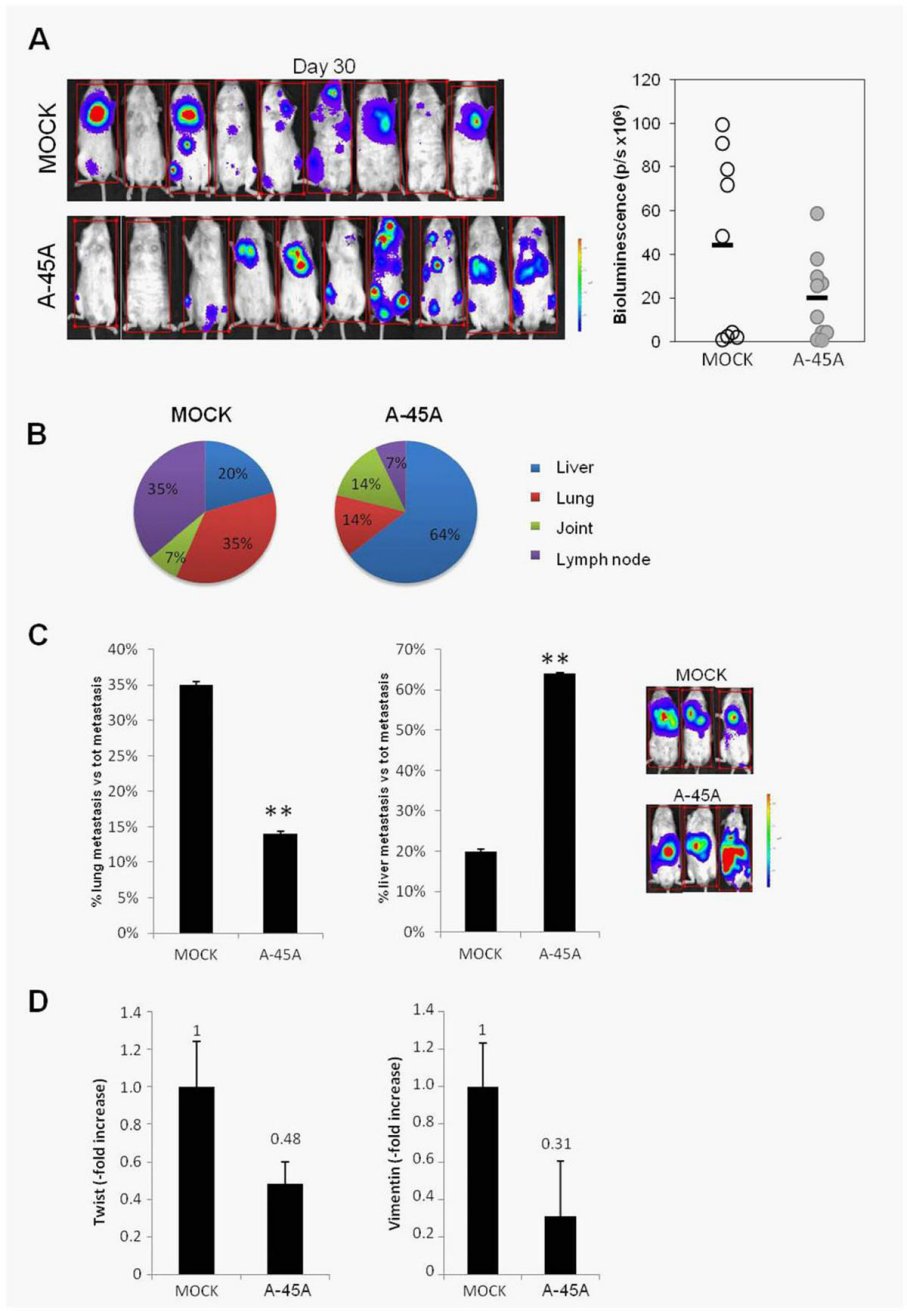
Effect of ncRNA on 45A down-regulation in an in vivo model of metastatic tumor **A**. Left panel represent Bioluminescence analysis of mouse 30 days after injection of SKNBE2-Mock and SKNBE2-Anti45A cells line. On the right panel, bioluminescent assay (p/s) of tumor distribution at the same interval time is shown. Empty circle: SKNBE2-Mock cells lines; grey circle: SKNBE2-Anti45A cells lines. **B**. Several organs were analyzed for the different distribution of tumor metastasis. Graphics represent the percentages of metastasis generate from mice injected with SKNBE2-Mock and SKNBE2-Anti45A cells lines. Analysis was performed using 10 mice for each group. **C**. Histogram represents the percentages of metastasis found in lung or liver, respectively, in mice injected with SKNBE2-Mock and SKNBE2-Anti45A cells line. Right images acquired with IVIS instrument shows three representative mice per groups. Data represent mean ± SD (**p < 0.01). **D**. Twist and Vimentin expression levels analyzed by Real-Time RT-PCR in Mock and 45A-downregulated cells. Data represent mean ± SD.

Besides our previous analysis of tumor nodules growth that took advantage of subcutaneously-injected mice xenografts, we decided to monitor *in vivo* the metastatic potential of 45A-downregulated nodules. To this aim, Mock and Anti45A-overexpressing cells were infected with a retroviral vector encoding the firefly luciferase gene to generate luciferase-positive cells. The development of metastasis and their growth rate were followed in the whole body using the IVIS technology. Thirty days after injection, total bioluminescence imaging (BLI) was lower in Anti-45A-injected mice as compared to control animals, even if the total number of metastasis was comparable in both groups (Figure 8A). Interestingly, after necroscopic analysis, we observed that mice injected with SKNBE2-Anti45A cells preferentially displayed metastasis within liver although, at a lesser extent, also lung, joints and lymph nodes were involved. On the contrary, mice treated with SKNBE2-Mock cells showed tumor spreading mainly in lung and lymph nodes and a minor contamination of liver and joints (Figures 8B and 8C).

Altogether these results demonstrate that the downregulation of 45A ncRNA leads to a decreased metastatic potential in tumor cells and to their reduced possibility to engraft in lung and lymph nodes tissues, most likely due to the alteration of cytoskeleton and adhesiveness. This view is also supported by the downregulation of Twist and Vimentin in 45A-downregulated cells, as resulting by Real-Time RT-PCR determination, which indicates the loss of mesenchymal traits acquired by malignant cells during the Epithelial-Mesenchymal Transition (Figure 8D). This alteration might also render 45A downregulated cells particularly adapted to engraft the liver forming hepatic metastasis.

## DISCUSSION

We recently reported the role of a pool of Pol III encoded ncRNAs as post-transcriptional regulators of several genes involved in both neurodegenerative and/or proliferative pathological conditions [2], [16]–[23]. Particularly, we observed that the expression level of one of these ncRNA, named 45A, regulates the malignant potential of neuroblastoma cancer cells *in vitro* and *in vivo* driving to dramatic changes of cancer cell phenotype that strongly affect cell proliferation control, short-term response to genotoxic stress and substrate adhesion capacity [2].

In the present work, we investigated, *in vitro* and *in vivo*, the effects of a stable downregulation of 45A ncRNA on tumor formation ability of NB cells. Our results show that the reduced expression of this transcript strongly decreases cell capability to form colonies in non-adherent conditions. This behavior is associated to a delay in mitotic entry of cells that poorly express 45A ncRNA, also demonstrated by an increased level of aneuploidy. Our data also show that the 45A ncRNA downregulation leads to a decreased cell survival consequent to an increased susceptibility to accumulate DNA damage. The characterization of this phenotype *in vitro* also evidenced remarkable changes in parameters associated to cytoskeleton structure that directly impact on carcinogenesis including cell morphology and migration potential as the downregulation of 45A ncRNA confers to the cell a less rounded shape (with a decreased stiffness and an increased flexibility) more prone to adhere to the substrate, less prone to migrate and less suitable to form colonies in methylcellulose, thus less efficient in the colonization of tissues and in the formation of metastasis.

These phenotypical traits are supported, at the genetic level, by the gene expression analysis, showing that a low expression of 45A ncRNA significantly affects the expression level of cell division- and morphology-related genes. All these aspects are in agreement with the slow-down of the proliferation (documented also by the reduced KI-67 level) and the increased compactness of tumor nodules composed by Anti-45A-expressing cells, as observed *in vivo*.

Of particular interest is the decreased expression of GTSE1 driven by 45A-downregulation. Indeed, GTSE1 is a negative regulator of p53 that can shuttle between the cytoplasm and nucleus. After DNA damage, GTSE1 accumulates in the nucleus, where it interacts with p53 and shuttles it out of the nucleus to promote its downregulation and recovery from the p53-induced G2 DNA damage checkpoint [24]. In addition, GTSE1 has been recently identified as microtubule-associated +TIP protein required for EB1-dependent cell migration. It directly interacts with microtubules in interphase and is enriched at growing microtubule plus ends through interaction with EB1. In this context it is very interesting to note that a positive relationship between GTSE1 protein levels and cell migratory capacity has been reported [6]. It should be also noted that GTSE1 is specifically expressed during S and G2 phases of the cell cycle and mainly localized to the microtubules. Several experiments demonstrated that it plays a role in the control of G2/M transition and in the regulation of p53 function [25]. Therefore, the above data strengthened our hypothesis that the 45A ncRNA downregulation affects cell morphology and its adhesion capability restricting the tumorigenic potential of cancer cells.

Interestingly, these cell phenotype changes drive to an altered tumor formation potential *in vitro* and *in vivo* as Anti-45 ncRNA expression in SKNBE2 tumor cell line is associated to the reorganization of the stromal tissue component in tumor nodules compatible with an impairment of cell-to-cell contact, adhesion and/or extracellular matrix deposition. Notably, the finding that the above mentioned phenotype changes can be observed also in a non-neuroblastoma cell suggests that the biological activity of 45A ncRNA is not restricted to this cell type. It is common knowledge that the fibrillar collagen network in tumor and normal tissues is different, due to remodeling of the extracellular matrix during the malignant character acquisition. In detail, normal tissues have more collagen than tumor tissues where a cap of collagen was observed at the periphery whereas within the tumor mass the distribution of collagen is heterogeneously spread [26]. In this context, our results suggest that the downregulation of 45A ncRNA inhibits SKNBE2 tumorigenic potential *in vivo*.

The analysis of the tumors induced *in vivo* strengthen the view that 45A ncRNA affects the genetic program of cell-cell contacts being 45A-downregulated nodules more compact, less proliferating and poorly expressing GTSE1, a panel of characteristics that, altogether, strongly support a different ability to metastasize. This is also shown by the distribution of the metastatic nodules shown by the Anti45A-injected mice. Thus, the deep investigation of the mechanisms that make 45A-downregulated cells prone to colonize the liver might disclose novel hints in the field of metastatization.

In conclusion, this work demonstrates that the expression level of 45A ncRNA influences the compactness of tumor nodules, their capability to colonize different tissues forming metastasis thus deserving further investigations with possible prognostic and/or therapeutic significance.

## MATERIALS AND METHODS

### Cell culture and transfection

HEK-293 (Human Embryonic Kidney) were maintained on Dulbecco's modified Eagles medium (DMEM, ECB7501L, EuroClone, Milan, Italy), 10% FBS (DE14-801F, Lonza, Milan, Italy), L-Glutamine (2 mM; EuroClone), and penicillin–streptomycin (100 U/ml/100 μg/ml; EuroClone). SKNBE2 (Human Neuroblastoma) cells were maintained on RPMI 1640 medium (ECB9006L, EuroClone), 10% FBS (DE14-801F, Lonza), L-glutamine (2 mM; EuroClone), penicillin–streptomycin (100 U/ml/100 μg/ml; EuroClone) (standard medium). HEK-293 cells were transfected using polyethylenimine (PEI; P3143, Sigma-Aldrich, Milan, Italy), SKNBE2 cells by a Microporator MP-100 (Digital Bio; NanoEnTek, Seoul, Korea; Labtech France) with pEGFP-N1 (Clontech, USA; restriction site used for molecular cloning procedures: AseI) as control (hereafter referred to as Mock) and pEGFP-N1-Anti-45A (hereafter referred to as Anti-45A). G418 (Geneticin; Invitrogen, Carlsbad, CA, USA) was used in culture medium as mean of selection up to 1 000 μg/ml, until resistant cells were identified. After selection, the cells were preserved in 200 μg/ml G-418 in standard culture conditions. The sequence of Anti45A is 5'-GGAAACAAACAAAGGGGAAAAATAAGATACGACCAAATTCATTATGATTTCACTTTTATGGTTAAATAGATTTCTAGCTAGA GCTGGAGTTGCTTCTCAATTAAACAGATATTTCTGGAGGGCCTGAAATCTGCCAAACCCTATACTAGTCTGTGAC TTGATTTCACCTGTTGATTGGGTGAATAGCTCCTTTTTTGCTTTGAGTTGGTTTAAAAAATTCTTAGCAGCAGCTCTCAG CATAATTTATTAGGCAAGAGTTTAGGAATGTTATTCCATGAAAGCTGCTTTCCTGCATGCTTGACGGGTAGCACCATGTCTGC-3'.

### Retrovirus production and infection

Retroviruses were prepared by calcium phosphate co-transfection of HEK-293 T cells mixing the retroviral expression vector pL-Luc-IH, encoding the firefly luciferase gene linked by a IRES sequence to the hygromycin resistance (Hygro R) gene [27], with the packaging vector pkat2ampac [28]. The retrovirus-containing supernatant was harvested 48 h post-transfection and used to infect target cells in the presence of 8 μg/mL Polybrene (Sigma-Aldrich). After 48 h, the transduced cell lines were selected with Hygromycin B 100 μg/mL (Sigma-Aldrich).

### Real time quantitative RT-PCR analysis

Total RNAs from samples were extracted using TRIzol reagent (Invitrogen) according to the manufacturer's protocol, DNAseI-digested and subjected to reverse transcription by Transcriptor High Fidelity cDNA Synthesis Kit (05081955001, Roche, Germany) following manufacturer's instructions. The total RNA from samples was measured by quantitative RT-PCR Real-Time using PE ABI PRISM@ 7700 Sequence Detection System (Perkin Elmer Corp./Applied Biosystems, Foster City, CA) and Sybr Green method following manufacturer's instructions. The sequences of forward and reverse primers were: 45A: 5’-CATCTATAATGGCTGAATTGGAA-3’ and 5’-ATGAACTTTCCAACAAATGTTGTT-3’; GTSE1: 5’-GCCACATGCTGGGGATGTGC-3’ and 5’-GCCCC GGGTGCTGTCAATGT-3’. For endogenous control, the expression of Glyceraldehyde 3 phosphate dehydrogenase (GAPDH) was examined. The sequences for human GAPDH primers were 5'-GAAGGTGAAGGTCGGAGTC-3' and 5'-GAAGA TGGTGATGGGATTTC-3'. Relative transcript levels were determined from the relative standard curve constructed from stock cDNA dilutions, and divided by the target quantity of the calibrator following manufacturer's instructions.

### Cell proliferation assays

For cell counting studies, cells were seeded at 1.5 × 10^5^ cells in 6 well plates, incubated in standard medium and counted with a hemocytometer after 48 hours.

### Cell cycle analysis

The cell cycle was analyzed after 70/30% ethanol/water fixation permeabilization with 1% Nonidet P-40 (Sigma-Aldrich) and labeling with propidium iodide (PI; Sigma-Aldrich). Samples were run on a FACSort cytofluorimeter (Becton Dickinson, Palo Alto, CA, USA), and cell cycle analysis was performed by the ModFit LT 3.0 computer program (Verity Software House, Topsham, ME, USA).

### cDNA synthesis and real time-PCR array

The cDNA for each RNA sample was obtained using RT^2^ First Strand kit (SABiosciences Corporation, QIAGEN Company, Frederick, MD) according to the manufacturer's instructions. Briefly, after genomic DNA elimination, the reverse transcription reaction was performed at 42°C for 15 min and then heated at 95°C for 5 min to inactivate the enzyme. The cDNA was mixed with RT^2^ SYBR green/ROX qPCR master mix (SABiosciences Corporation) and 25 μl aliquots were loaded into each well of the RT^2^ Profiler PCR Array (Cat. No. PAHS-020, SABiosciences Corporation). The PCR array was designed to study the profile of 84 human cell-cycle-related genes. PCR array experiments were performed on an Eppendorf Realplex^4^ epgradient S Mastercycler^®^. Conditions for amplification were as follows: 1 cycle of 10 min at 95°C followed by 40 cycles of 15 s at 95°C and 1 min at 60°C. The PCR array data were analyzed by PCR Array Data Analysis web-based software (SABiosciences Corporation).

### Colony forming assay

Cells were seeded at 400 cells in 60 mm dish, 3 dishes per dose, for the determination of cell survival measured as colony forming ability. After 8–10 days, the dishes were fixed and colonies counted.

### MN assay

The day before treatment, cells were seeded in 35-mm dishes coverslips. Cells were cultured at densities of 3×10^5^ cells/coverslip, using conditions ensuring that cells were actively growing after 1 h treatment MMS, depending from the intensity of the lamp. At the appropriate harvest time, cells were rinsed and exposed to a hypotonic shock *in situ* using Iskandar solution (0.9% NaCl/75 mM KCl; 9:1). Cells were then fixed with MeOH/HOAc (3:1) and stained with Giemsa. All slides including those of positive and solvent controls were coded before analysis and scored blindly for the evaluation of MN by two independent observers. The criteria used for identifying MN fulfil those recommended by the HUMNwork [25]: (1) area <1/3 the main nucleus area; (2) no overlapping with the nucleus (distinct borders); and (3) same aspect as the chromatin. MMS was tested at doses 0.05 mM and 0.1 mM; UV doses were 5 J/m^2^ and 10 J/m^2^.

### Methylcellulose colony formation assay

Clonogenic assay was performed using a methylcellulose medium consisting of RPMI 1640 with 0.9% methylcellulose (Methocult H4100; StemCell Technologies, Vancouver, BC, Canada), 10% FBS, 100 U/ml penicillin/streptomycin and 2 mM L-glutamine. Cells were plated at a density of 400 cells in 2 ml volume of methylcellulose medium in humidified 6-well plates. For each assay, cells were plated in triplicate. Colonies were counted at 14 days after plating.

### Cell migration assay

Cell migration was analysed by the technique of real-time migration monitoring using the CIM-Plate 16 and xCELLigence System RTCA DP Instrument (Roche). The day before migration assay, cells were plated in starvation medium (medium with FBS 0.5%). After 24 hr, 4×10^4^ cells, resuspended in 100 μl of serum-free medium, were seeded in the upper chamber of a CIM-Plate 16. The upper chamber was then placed on the lower chamber of the CIM-Plate 16 containing growth medium supplemented with 20% FBS as an attractant, or without FBS (negative control). Time-dependent cell migration was monitored over a period of up to 60 hours, and experimental results were analyzed using RTCA Software 1.2 considering the interval of 0-6 hours.

### Cell adhesion assay

This protocol was adapted from Mc Clay et al., 1981 [29]. Cells were plated at 1.5×10^5^ cells/well in a 24-well tissue culture plate; 3 h after cell plating, the multi-well plate was centrifuged twice in inverted position (well bottom placed up in a swing-out rotor) at 1 000 rpm for 30 s. The cells still adherent to the wells were then fixed in 3.7% paraformaldehyde in PBS, stained with methylene blue and counted (each well was divided in quadrants and 3 quadrants/well were counted). Each value represents the mean ± SD of three independent wells.

### Monolayer wound healing assay

Cells were grown to full confluence in 6-well plates in their growth media and were scratched with a 1 000 μL sterile pipette tip. Medium was change to remove detached cells and six pictures for well were acquired at 4x magnification after 24, 48 and 72 hours, using an EVOS fl Digital Inverted Fluorescence Microscope (Advanced Microscopy Group, WA, USA). The open wound area (24 h, relative to 0 h values) was quantified with TScratch software. Data represent mean ± SEM of 6 replicates. Results are representative for 3 independent experiments.

### Western blot

Western blotting was performed with a SDS-PAGE Electrophoresis System as described previously [30]. Briefly, protein samples were electrophoresed on 12.5% polyacrylamide gels under reducing conditions; blotted to nitrocellulose membrane; and sequentially probed with antibodies. The antibodies used were: rabbit polyclonal anti-GTSE1 (ab103232, Abcam, Cambridge, UK), mouse monoclonal anti-β-Actin antibody (A5441, Sigma-Aldrich), anti-rabbit IgG (A0545, Sigma-Aldrich), anti-mouse IgG (A0168, Sigma-Aldrich). The densitometric analysis of protein bands was performed using the ImageJ software system.

### Ethics statement

Investigation has been conducted in accordance with the ethical standards. The care and the use of the animals were in compliance with laws of the Italian Ministry of Health and the guidelines of the European Community. All experiments involving animals were reviewed and approved by the licensing and ethical committee of the AUO San Martino IST, Genoa, Italy, and by the Italian Ministry of Health. Efforts were made to minimize animal stress/discomfort.

### *In vivo* tumorigenicity assay

Homozygous NOD-SCID (NOD.CB17-Prkdcscid) mice were purchased from the Jackson Laboratory (Bar Harbor, MA, USA) and housed under specific pathogen-free conditions. Mice were used between 5 and 8 weeks of age. All animals were bred and maintained at the institution's animal facility of the National Institute for Cancer Research, Genoa, Italy.

A cell suspension of SKNBE2_Mock-, SKNBE2-Anti45A (3×10^6^ cells) in PBS was subcutaneously injected into NOD/SCID mice. Ten mice for each cell line were divided in 2 groups: i) mice injected with Mock cells (n=5); ii) Mice injected with Anti45A-overexpressing cells (n=5). Mice were observed weekly for the appearance sites: tumor size was measured daily with calipers in all groups and tumor volume was calculated by the formula length^2^ × width × π/6. Mice injected *s.c*. were sacrificed when the tumor size reached 2 000 mm^3^ or greater. To calculate the tumor development function by the polynomial interpolation, we used the average of the daily measures when each mouse had the tumor mass. For each group the averaged progression rate by the linear regression was calculated. In order to detect if the expression of Anti-45A transcript (and the consequent downregulation of 45RNA expression) persists after the injection of cells in mice, we isolated SKNBE2 cells from tumor nodules, by Trypsin/Collagenase digestion, and measured the 45A expression levels by Real-Time RT-PCR, confirming the downregulation of the ncRNA (data not shown).

### Histological analysis

For histological examination, tumors derived from each experimental group were surgically removed and fixed in 4% neutral-buffered formalin, dehydrated and embedded in paraffin using standard histological techniques. Serial 5 μm sections were cut and stained with Mallory's trichrome for detection of collagen fibrils (deep blue), nuclei and neuroglia fibrils (red). Images were captured by transmitted light microscopy using a Zeiss Axiovert 200M microscope equipped with a Zeiss Axio-Cam MRc color chilled 3CCD camera (Zeiss, Wetzlar, Germany).

### DAB-Immunohistochemical staining and quantification

Immunochemistry detection of KI-67 and GTSE1 was conducted on sections of 4% paraformaldehyde (PFA) fixed paraffin-embedded tumors explanted from mice. Antigen retrieval was done with 6.0 pH citrate buffer in a microwave oven. The sections were immunostained using rabbit anti-KI-67 (SP6 NB600-1252 Novus Biologicals), anti-GTSE1 (ab103232, Abcam), overnight at 4°C. The antibody complex was revealed with Rabbit EnVision+ System-Peroxidase (Dako) and Diaminobenzidine peroxidase reaction (DAB). The sections were counterstained with modified Mayer hematoxylin and mounted in Glycerol gel (Dako).

For quantification of DAB-Immunohistochemical staining with GTSE1, 8 randomly chosen microscope fields from each SKNBE2-Mock and SKNBE2-Anti45A tumor nodule sections were captured at 60x magnification and the proportion of GTSE1 positive areas determined using ImageJ software. Images were converted in HSB and threshold was manually adjusted to localize DAB-stained areas of interest. The number of pixels within the hue range set was determined and expressed as a percentage of the total selected area (yellow line).

For KI-67, immunohystochemical staining quantification 10 images per sample representing central and peripheral tumor areas were acquired using 60X objective. The percentage of DAB-stained nuclei of the total nuclei (DAB and hematoxylin-stained) was calculated using the application, named ImmunoRatio, that calculates the percentage of positively stained nuclear area (labeling index) by using a color deconvolution algorithm for separating the staining components. From 5 SKNBE2-Mock and 5 SKNBE2-Anti45A samples, 10 images per sample representing central and peripheral tumor areas were acquired using 60X objective.

### Metastatic neuroblastoma mouse model and luciferase detection

Mice were purchased from Charles River (CALCO Italy) and housed under specific pathogen-free conditions. Six-week-old male and female NOD/SCID mice were intracardiac injected with pmeLUC-SKNBE2-Mock and SKNBE2-Anti45A cells (1×10^5^ cells/mouse). BLI was performed evaluating the stably pmeLUC-transfected NB cell, by highly sensitive, cooled CCD camera mounted in a light-tight specimen box (IVIS; Xenogen, Waltham, MA, USA). Tumor growth was assessed weekly by bioluminescence imaging. Briefly, 10 min before every acquisition, animals were intraperitoneally injected with the substrate D-luciferin (Promega, Madison, WI) and anesthetized with 1–3% isofluorane (Veterinaria Esteve, Alcazar De San Juan, Spain). Mice were then placed onto the warmed stage, inside the light-tight camera box, with continuous exposure to 1–2% isofluorane. Bioluminescence intensity (BLI) is expressed as photons per second (p/s).

### Evaluation of cell mechanical properties

AFM measurements were performed by using a Nanowizard III (JPK Instruments, Germany) mounted on an Axio Observer D1 (Carl Zeiss, Germany) inverted optical microscope. V-shaped silicon nitride cantilevers (NP, Bruker, USA), with a nominal spring constant ranging from 0.06 N/m and pyramidal tip with typical curvature radius of 20 nm were used. The actual spring constant of each cantilever was determined *in situ*, using the thermal noise method [31].

The experiments were performed on living cells, working in RPMI 1640 medium (ECB9006L EuroClone), 10% FBS (DE14-801F, Lonza), L-glutamine (2 mM; EuroClone), penicillin–streptomycin (100 U/ml/ 100 μ g/ml; EuroClone) (standard medium) and maintaining the temperature constant at 37°C. AFM experiments were performed in less than 1 h, in order to preserve cell viability. Force maps (32×32 Force distance (FD) curves) were acquired on small area of 5×5 μm^2^ on the central part of the cell by using the QI mode option (JPK Instruments, Germany) that allowed for a fast acquisition of a high number of FD curve. The tested areas were selected directly on the optical images; the exact superimposition between optical images and the AFM tip positions was obtained by using the Direct Overlay routine of the AFM acquisition software (JPK Instruments, Germany). The maximum force applied on the sample was 350 pN in all the tested points, FD curves length was varying between 1.0 and 1.5 μm, the tip velocity maintained constant to 25 μm/s.

FD curves were converted in force *vs*. tip-sample separation curves (FI). The Hertz's model was used to calculate the Young's (*E*) modulus of cells. The best fit between the experimental data and theoretical model of a non-deformable pyramidal indenter provided the value of E. The Poisson's ratio was considered 0.5. Maximal indentations of 600 nm were taken in to account by the fitting procedure, in order to minimize the effect of the presence of a rigid substrate on the result.

### Statistical analysis

Results are expressed as mean ± Standard Deviation. Statistical significance of observed differences among different experimental groups was calculated using a two-tailed unpaired Student's t test. A P value of less than 0.05 was considered to be statistically significant. In the figures, * and ** indicate statistical significance at p < 0.05 and 0.01, respectively. The statistical calculations were performed with GraphPad Prism 6.0 for Windows (GraphPad Software, La Jolla, CA, USA).

## SUPPLEMENTARY MATERIALS FIGURES AND TABLES



## Abbreviations

ncRNA: non-coding RNA
pol: polymerase
AD: Alzheimer disease
Aβ: amyloid beta
APBB2: amyloid beta precursor protein-binding, family B, member 2
APP: amyloid precursor protein
GTSE1: G2 and S phase-expressed-1
MMS: methylmethanesulfonate
MN: micronuclei
FR: fragmented nuclei
MultiN: multinucleated cells
BER: base excision repair
AFM: atomic force microscopy
RT-PCR: Reverse Transcriptase-Polymerase Chain Reaction
BLI: total bioluminescence imaging
IF: immunofluorescence
GFP: green fluorescent protein
DAB: Diaminobenzidine
