# Supplementary material for: The inhibition of 45A ncRNA expression reduces tumor formation, affecting tumor nodules compactness and metastatic potential in neuroblastoma cells
Source: Oncotarget. 2016 Dec 24;8(5):8189–205. doi: 10.18632/oncotarget.14138 (PMC5352393; doi:10.18632/oncotarget.14138)
Supplement: Supplementary file 1 [file oncotarget-08-8189-s001.pdf]

The inhibition of 45A ncRNA expression reduces tumor formation, affecting tumor nodules compactness and metastatic potential in neuroblastoma cells

SUPPLEMENTARY DATA

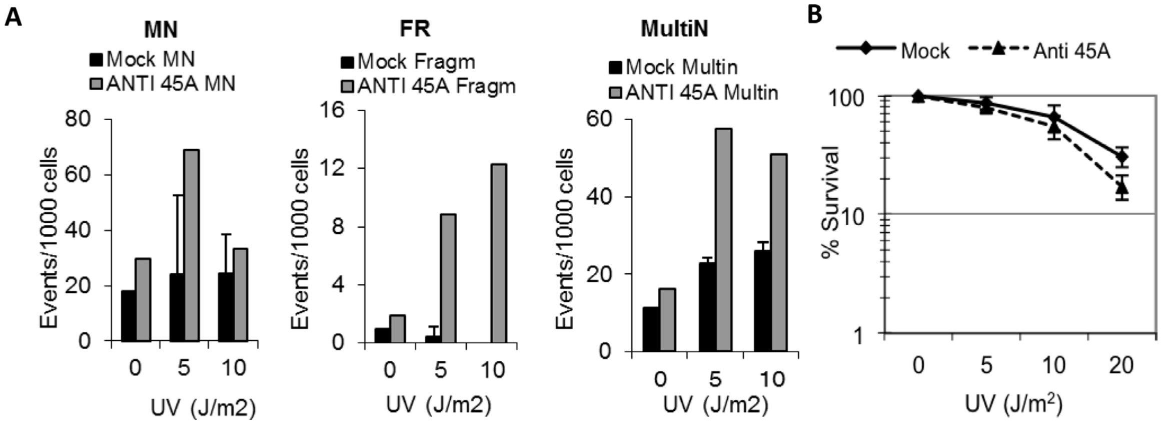

Supplementary Data 1: A. Evaluation of Micronuclei (MN), Fragmented cells (FR), Multinucleated cells (MultiN) and **B.** survival rate in HEK-293-Mock and HEK-293-Anti45A before and after UV radiation treatment. Data represent mean ± SD.

Up-regulated Genes				
**Gene name**	**Gene Symbol**	**Genebank Acc.**	**Fold Reg**	**Function**
MAD2 mitotic arrest deficient-like 2 (yeast)	MAD2L2	NC_000001.10	35.85	Component of the mitotic spindle assembly checkpoint that prevents the onset of anaphase until all chromosomes are properly aligned at the metaphase plate. GENECARDS
Minichromosome maintenance complex component 3	MCM3	NC_000006.11	51.41	Required for DNA replication and cell proliferation. GENECARDS
Nibrin	NBN	NC_000008.10	35.11	Component of the MRE11/RAD50/NBN (MRN complex) which plays a critical role in the cellular response to DNA damage and the maintenance of chromosome integrity. The complex is involved in double-strand break (DSB) repair. GENECARDS
Retinoblastoma-Like 2 (p130)	RBL2	NC_000016.9	75.27	May act as a tumor suppressor. GENECARDS
Ubiquitin-like modifier activating enzyme 1	UBE1	NC_000023.10	43.83	It marks cellular proteins for degradation. It may also function in DNA repair. GENECARDS
**Down-regulated genes**				
Anaphase promoting complex subunit 2	ANAPC2	NC_000009.11	- 1.16	Promotes metaphase-anaphase transition by ubiquitinating its specific substrates such as mitotic cyclins and anaphase inhibitor. GENECARDS
CDC28 protein kinase regulatory subunit 1B	CKS1B	NC_000001.10	-3.60	CKS1B protein binds to the catalytic subunit of the cyclin dependent kinases and is essential for their biological function. GENECARDS
Growth arrest and DNA-damage-inducible. alpha	GADD45A	NC_000001.10	-2.23	It stimulates DNA excision repair in vitro and inhibits entry of cells into S phase. GENECARDS
G-2 and S-phase expressed 1	GTSE1	NC_000022.10	-55.18	Microtubule protein acting in cell migration regulation and involved in metastasis (Scolz et al., 2012)
HUS1 checkpoint homolog (S. pombe)	HUS1	NC_000007.13	-1.13	It plays a major role in DNA repair. GENECARDS
MRE11 meiotic recombination 11 homolog A (S. cerevisiae)	MRE11A	NC_000011.9	-3.57	Component of the MRN complex, which plays a central role in double-strand break (DSB) repair. GENECARDS

Supplementary Data 2: Table: Downregulated and upregulated genes involved in cell cycle control (by cell cycle-specific Real-Time low density array) in HEK-Anti45A respect to Mock cells.

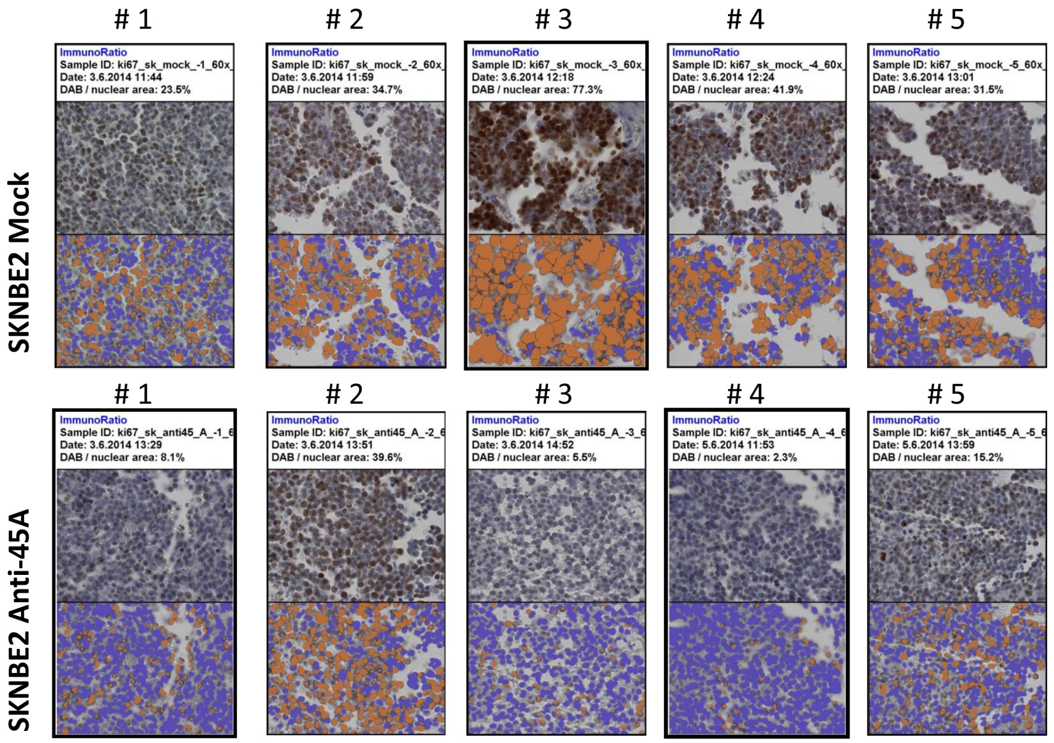

Supplementary Data 3: Immunohistochemical distribution and quantification of KI-67 in SKNBE-Anti45A and SKNBE-Mock tumor nodules. Representative randomly chosen microscope fields at high magnification (60x) KI-67 immunohistochemical (IHC) stain from SKNBE-Mock and SKNBE-Anti45A tumor nodule sections from different mice, analised using digital image analysis (ImmunoRatio). In upper level original images (IHC stain); in lower level pseudo-colored images showing staining components (Dab staining nuclei in orange).

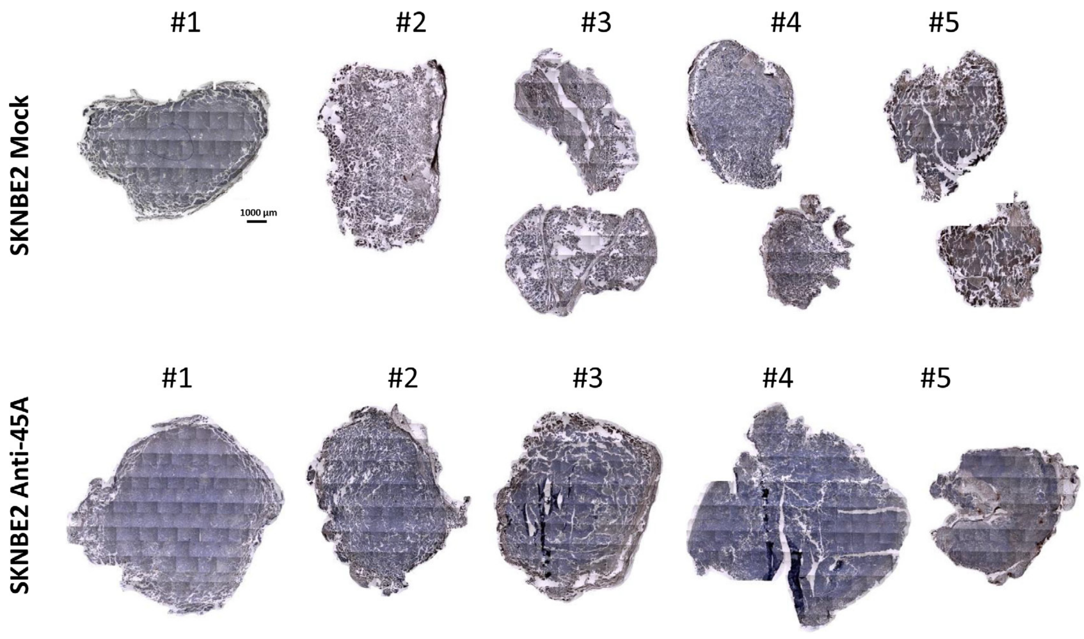

Supplementary Data 4: GTSE1 immunohistochemical (IHC) stain from SKNBE2-Mock and SKNBE2-Anti45A tumor nodule sections. Reconstituted GTSE1 stained tumor nodule sections obtain from mice inoculated with SKNBE2-Mock or SKNBE2-Anti45A.

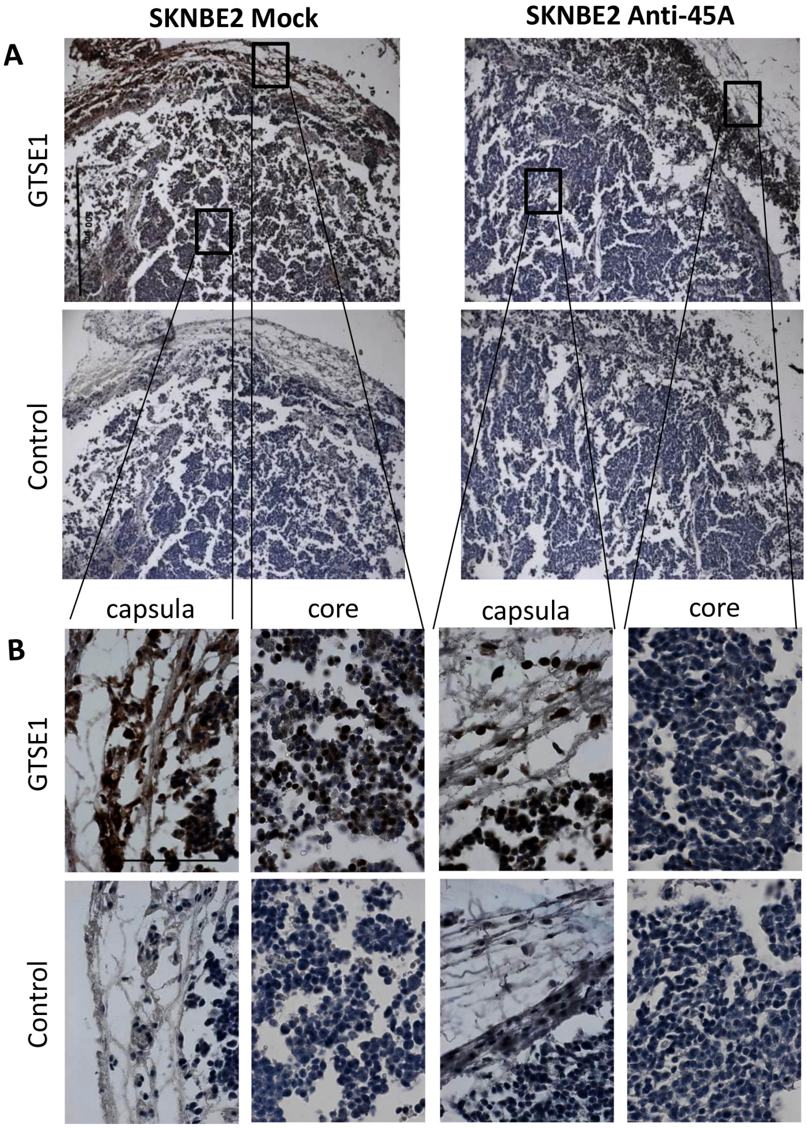

Supplementary Data 5: Immunohistochemical analysis of GTSE1 expression and distribution in SKNBE2-Mock and -Anti45A tumor nodules. A. Representative 10x magnification (scale bar: 500 µm) and **B.** 60x magnification (scale bar: 100 µm) of KI67 immunohistochemical (IHC) stain from SKNBE2-Mock and SKNBE2-Anti45A tumor nodules (scale bar: 500 µm).

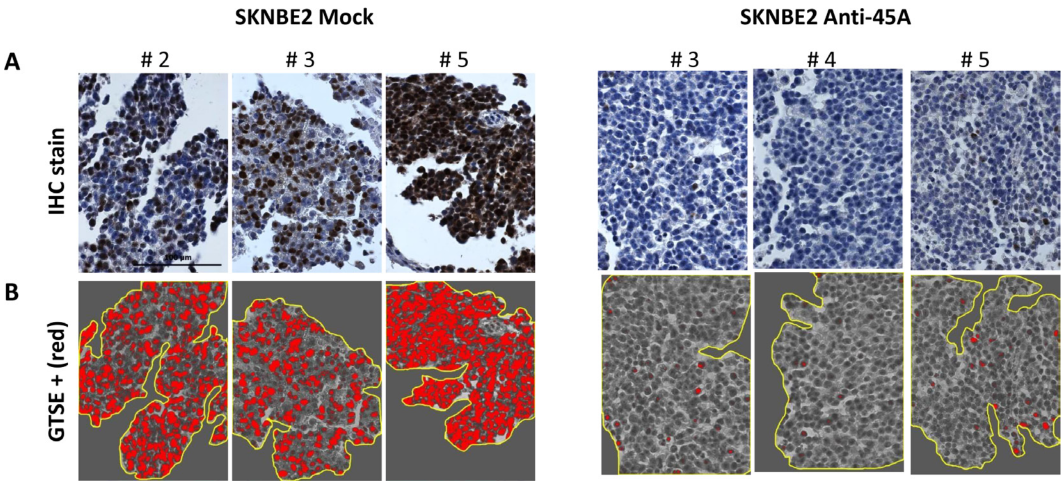

Supplementary Data 6: Immunohistochemical distribution and quantification of GTSE1 in SKNBE2-Anti45A and SKNBE2-Mock tumor nodules: A. Representative randomly chosen microscope fields at high magnification (60x) of GTSE1 immunohistochemical (IHC) stain from SKNBE2-Mock and SKNBE2-Anti45A tumor nodule sections from different mice (Scale bar=100 µm). **B.** Images converted in HSB and threshold manually adjusted to quantify GTSE1 positive areas using Image J (Scale bar=100 µm).
